# Identification of Mental Health Disorder Symptoms, Predisposing Factors and their Impact on Quality of Life in Young Hypertensive Patients: A Multicenter Longitudinal Study

**DOI:** 10.2174/0117450179470919260727062056

**Published:** 2026-07-29

**Authors:** Elmeida Effendy, Mustafa Mahmud Amin, Vita Camellia, Rika Sarfika, Christantie Effendy, Tiasarah Aretha Sitepu, Dewi Gita Maharani

**Affiliations:** 1 Department of Psychiatry, Faculty of Medicine, Universitas Sumatera Utara, North Sumatera, Indonesia; 2 Department of Mental Health and Community Nursing, Faculty of Nursing, Andalas University, Padang, West Sumatra, Indonesia; 3 Department of Medical Surgical Nursing, Faculty of Medicine, Public Health, and Nursing, Gadjah Mada University, Sleman DI Yogyakarta, Indonesia

**Keywords:** Young hypertension, Mental health, Quality of life, Multicenter longitudinal study, Psychological distress, Anxiety

## Abstract

**Introduction:**

Mental health problems in young adults with hypertension are often underrecognized, despite their potential impact on quality of life and long-term outcomes. Evidence in younger populations remains limited, particularly in longitudinal and multicenter settings.

**Objective:**

To examine the relationship between hypertension duration, mental health symptoms, and quality of life among young adults, as well as to explore psychosocial stressors identified through qualitative inquiry.

**Methods:**

This multicenter longitudinal study recruited patients aged 18–45 years with hypertension from outpatient clinics across several Indonesian cities. Participants were assessed at baseline using the Depression Anxiety Stress Scale-21 (DASS-21) and the WHO Quality of Life-BREF (WHOQOL-BREF).

Hypertension duration was recorded as a continuous variable. A subset of participants underwent semi-structured interviews to explore psychosocial stressors. Quantitative data were analyzed using correlation analysis, while qualitative data were analyzed thematically.

**Results:**

A total of 791 participants were included in the study, with a mean age of 33 ± 10.14 years, and women slightly outnumbered men. This study found that a longer duration of hypertension was associated with worse overall quality of life (r = -0.45) and lower psychological well-being (r = -0.40). Notable correlations also emerged between hypertension duration and higher levels of anxiety (r = -0.42), depression (r = -0.38), and stress (r = -0.30), while socioeconomic and psychosocial stressors emerged as major contributing factors.

**Discussion:**

This multicenter longitudinal study suggests a direct link between early-adulthood hypertension and subsequent psychological distress, particularly anxiety and depressive symptoms, as well as lower quality of life with aging. Recent findings underscore that hypertension in young individuals extends beyond cardiovascular risk and may also impose a substantial mental health burden. Evidence identifying socioeconomic and psychosocial stressors as key predisposing factors further strengthens the rationale for incorporating routine mental health screening and psychosocial interventions into standard hypertension management protocols, thereby enhancing patients’ quality of life and potentially improving long-term clinical outcomes.

**Conclusion:**

In this multicenter longitudinal cohort, a longer duration of hypertension was associated with greater psychological distress and poorer quality of life among young adults. These findings support the integration of routine mental health screening into hypertension care.

## 1 INTRODUCTION

Hypertension represents a critical global public health concern and is a major contributor to morbidity and mortality, accounting for nearly 30% of all cardiovascular deaths. Indian studies have also highlighted the widespread impact of depression, affecting approximately 49% of the population. Notably, significant depressive symptoms were reported in 53.4% of hypertensive individuals, 44.6% of those with pre-hypertension, and 44.6% of those with stage II hypertension [1, 2].

Hypertension is a serious medical condition that substantially increases the risk of cardiovascular disease and its severe complications, including myocardial infarction and stroke. In parallel, mental health disorders, such as depression and anxiety, are among the leading causes of disability in adults and frequently impair physical health and overall well-being [3]. Although cardiovascular risk calculated using current guidelines may remain low in young adults due to their age, hypertension is still associated with an increased lifetime risk of cardiovascular disease in this population [4].

Although these conditions are typically studied in isolation, emerging research increasingly demonstrates a clear association between mental health and hypertension among young adults. Depression and anxiety are among the most common mental disorders; however, studies examining the relationship between depression and hypertension have produced inconsistent findings, and the exact nature of this association remains controversial [5].

Adulthood represents a pivotal period for preventive and therapeutic efforts; however, hypertension during this stage often remains silent, resulting in many individuals being diagnosed only after the condition has progressed and treatment has been delayed. At the same time, mental disorders are frequently overlooked or inadequately treated, partly due to persistent stigma and the limited availability or accessibility of appropriate mental health care. Furthermore, adults commonly face economic pressures, such as unstable income, job-related stress, and the challenges of navigating complex health systems, all of which may increase vulnerability to both hypertension and mental health problems [6]. Individuals with stronger social support are often less likely to seek mental health services independently, yet they tend to accept help more readily when it becomes necessary compared to those without adequate support [7].

The relationship between anxiety and hypertension has been widely studied in older adults, but evidence among younger and middle-aged populations remains comparatively limited. Some studies have reported positive associations between anxiety and hypertension, although most were cross-sectional in design. Others have found no association or even an inverse relationship. In addition, many studies use varying methodologies to define and classify anxiety and hypertension in young and middle-aged adults, which may contribute to the inconsistent findings [8].

Findings from various studies emphasize the importance of a holistic approach in managing mental health among young adults suffering from hypertension [9]. Moreover, studies on health literacy indicate that psychological counseling and health education are essential to improve self-management competence and adaptive coping among hypertensive patients [10].

## 2 METHODS

The purpose of this multicenter longitudinal study was to investigate mental health symptoms, associated risk factors, and their impact on quality of life among young adults aged 18–45 years diagnosed with hypertension. Participants were recruited from outpatient clinics at several hospitals over a 6-month period. A total of 791 eligible participants were recruited across all study centers using a consecutive sampling approach. Eligible participants were 18–45 years old, had been diagnosed with hypertension for at least 6 months, and were willing to provide written informed consent. Patients with a psychiatric diagnosis before the onset of hypertension and those unable to complete the assessments due to cognitive or physical impairment were excluded.

Data collection was conducted in two phases. In the first phase, participants completed two well-validated questionnaires: the Depression Anxiety Stress Scale-21 (DASS-21), which measures symptoms of depression, anxiety, and stress, and the World Health Organization Quality of Life Instrument (WHOQOL-BREF), which evaluates quality of life across four domains: physical health, psychological well-being, social relationships, and environment. These self-report questionnaires were distributed and collected during clinic visits.

Semi-structured interviews were then administered to all participants to explore their mental health status further. The interviews aimed to corroborate and validate the information obtained from the questionnaires, as well as to collect qualitative data related to risk factors for mental health problems, including family history of mental illness, socioeconomic conditions, and personal coping strategies. The sessions were conducted by trained interviewers in a private setting to protect confidentiality and encourage open dialogue.

All data were anonymized and analyzed using a mixed-methods approach. Quantitative data from the questionnaires were analyzed using correlation analysis to assess relationships among hypertension duration, mental health symptoms, and quality-of-life scores. The results are presented as correlation coefficients (r), with corresponding *p*-values and 95% confidence intervals. The 95% confidence intervals for the correlation coefficients were estimated using Fisher’s z transformation. Qualitative data obtained from the interviews were analyzed using thematic analysis to identify recurring patterns and contextual factors [11].

## 3 RESULTS

The respondent profile reflects a diverse and representative sample of young adults with hypertension. Participants were aged between 18 and 45 years, with a mean age of 33 ± 10.14 years. Female participants slightly outnumbered male participants. Detailed sociodemo-graphic characteristics are presented in Table **1**.

In terms of educational background, nearly half of the participants had completed high school (49.8%), followed by those with a bachelor’s degree (27.8%). The occupational distribution was varied, with the largest proportion of participants being unemployed (21.4%), followed by professionals (18.1%) and students (15.2%). Approximately 69.5% of participants were married.

Longer duration of hypertension was significantly associated with poorer quality of life across multiple domains of the WHOQOL-BREF. The strongest negative correlation was observed for overall quality of life (r = -0.45, 95% CI: -0.51 to -0.39, *p* < 0.001), indicating that prolonged exposure to hypertension is linked to a substantial decline in perceived quality of life.

Similarly, psychological health showed a moderate negative correlation (r = -0.40, 95% CI: -0.41 to -0.29, *p* < 0.001). The environment domain (r = -0.30, 95% CI: -0.36 to -0.24, *p* < 0.001) and social relationships domain (r = -0.25, 95% CI: -0.32 to -0.18, *p* < 0.001) also demonstrated statis-tically significant, although weaker, associations (Table **2**).

These findings indicate that a longer duration of hypertension is consistently associated with poorer quality of life across physical, psychological, environmental, and social domains.

Significant associations were also observed between the duration of hypertension and psychological distress, as measured by the DASS-21.

A moderate negative correlation was found for anxiety (r = -0.42, 95% CI: -0.48 to -0.36, *p* < 0.001), indicating that a longer duration of hypertension was associated with higher anxiety symptom scores. Similarly, depression showed a moderate negative correlation (r = -0.38, 95% CI: -0.44 to -0.32, *p* < 0.001), while stress demonstrated a weaker but still statistically significant association (r = -0.30, 95% CI: -0.36 to -0.24, *p* < 0.001), (Table **3**).

Given the scoring direction of the DASS-21, these negative correlations indicate that a longer duration of hypertension is associated with increased severity of psychological distress.

Overall, these findings highlight the harmful impact of chronic hypertension on mental health and emphasize the need for a multidisciplinary management approach that addresses both physical and psychological well-being over the long term. Consistent with the findings, Crepaldi *et al.* reported that comorbid mental disorders were associated with poorer quality of life in young adults with hypertension [12].

## 4 DISCUSSION

This study investigated the influence of childhood financial adversity on cardiovascular risk in young adulthood, focusing on family conflict and psychological distress as key mediating factors. The findings suggest that childhood socioeconomic deprivation may contribute to an increased likelihood of hypertension through psychological distress. In addition, exposure to family conflict during childhood was significantly associated with higher blood pressure in adulthood.

Childhood exposure to low socioeconomic conditions is a major risk factor for adult morbidity and mortality. Children from low-income families may show early signs of cardiovascular reactivity, including elevated arterial blood pressure and prehypertension. Stressful family relationships and parent–child conflict have also been associated with hypertension and prehypertension in children. Psychological factors have been widely examined as potential mediators in the relationship between socioeconomic status and health outcomes in both children and adults. Chronic exposure to poverty-related stress during childhood may contribute to anxiety and depression, which can increase physiological arousal and subsequently raise the risk of hypertension and cardiovascular disease later in life [13].

Previous studies have examined several psychological factors associated with cardiovascular health, including psychosocial stress, coping strategies, early-life stress, adverse childhood experiences, attachment patterns, anxiety, and depression. These stress-related factors appear to be more prevalent among patients with coronary disease than in the general population. Stressors may contribute to the etiology of cardiovascular disease by affecting neuroendocrine, autonomic, and cardiovascular regulation. Certain brain structures involved in autonomic and cardiovascular responses to stress play an important role in modulating these physiological pathways [14].

Early family instability during childhood has been linked to a higher likelihood of hypertension in adulthood, and this association may occur through indirect pathways across the life course. Adverse early-life environments, such as limited socioeconomic resources and poor psychosocial adjustment, may contribute to psychological and behavioral vulnerabilities that persist into adulthood. These vulnerabilities may later manifest as higher body mass index, poorer mental well-being, and unhealthy lifestyle behaviors or circumstances. Collectively, these psychological and metabolic factors may help explain how chronic, low-intensity stressors during early life contribute to long-term cardiovascular dysregulation and increased hypertension risk in adulthood [15].

Recent research has focused on the association between adverse childhood experiences (ACEs) and changes in blood pressure during adulthood, to clarify the underlying mechanisms and support early prevention and intervention strategies [16]. Hereditary factors and hyperactivity of the sympathetic nervous system are considered important mechanistic pathways in this association [17]. Blood pressure regulation is also influenced by genetic, environmental, and lifestyle factors. Among these, growing evidence suggests that ACEs are significant risk factors for the development of hypertension [18].

The prevalence of hypertension among older adults has also increased over time in several Indonesian cities, including Yogyakarta, Padang, and Medan, with nearly 40% of cases occurring among individuals aged 65 years and older. Although awareness and treatment of hypertension have improved, blood pressure control remains suboptimal in this population. Hypertension management in older adults should consider vascular aging and cardiovascular remodeling, which are commonly associated with aging and long-standing hypertension. The high burden of comorbidities also requires individualized decision-making regarding target blood pressure levels and the selection of antihypertensive medications. In addition, frailty and cognitive impairment may affect drug tolerance and increase the risk of orthostatic hypotension, syncope, falls, kidney injury, and electrolyte disturbances. Therefore, careful patient evaluation is necessary before initiating therapy.

A recent prospective study found that childhood family income has long-lasting effects on adult cardiovascular health, including key indicators, such as blood pressure, by influencing the socioeconomic context in which children grow up. Higher family income during childhood was associated with better cardiovascular health outcomes in adulthood, suggesting that long-term exposure to more advantaged environments can mitigate cardiovascular disease risk factors, including hypertension. These findings support the notion that socioeconomic disadvantage early in life can have sustained implications for cardiovascular health, consistent with models in which early life stress and economic hardship contribute to adult hypertension risk through psychosocial and environmental pathways [19].

Based on the data presented in the frequency distribution table, identification of mental health disorder symptoms, predisposing factors, and Impact on quality of life in young hypertensive patients in multicenter studies, such as Yogyakarta, Padang, Medan, and other cities, shows certain characteristics of the participants. This study aimed to examine the correlation between demographic variables, mental health, and hypertensive. A key finding was the significant negative correlation between the WHOQOL-BREF and DASS-21, indicating stronger mental health and lower hypertension.

These findings underscore the mental health burden in young hypertensive patients, aligning with recent evidence from tertiary care settings that psychological distress such as depression and anxiety significantly impairs cognitive function and overall quality of life, necessitating routine mental health screening alongside hypertension management [20].

The WHOQOL-BREF result indicated that the more patient suffering hypertensive, the poorer their quality of life was (- 0.45) compared to psychological health (-0.40), physical health (-0.35), environment (-0.30), and social relationships (-0,25). And for the DASS-21 with hypertension results, the more patient suffering hypertensive, the higher their anxiety level was compared to depression and stress.

These findings highlight the need to prioritize mental health care in young patients with hypertension. Given the bidirectional relationship between hypertension and mental health disorders, management should adopt a more comprehensive, integrated approach. Future studies should evaluate interventions that embed mental health support within hypertension care.

## 5 STUDY LIMITATIONS

This study has several limitations. First, its observational and correlational design prevents any causal conclusions about the links among hypertension duration, mental health symptoms, psychosocial stressors, and quality of life. Second, participants were recruited from outpatient clinics in several Indonesian cities, which may reduce the generalizability of the results to other regions, particularly rural settings, people who are not routinely engaged in healthcare, and different healthcare systems. Third, mental health symptoms, psychosocial stressors, and quality of life were measured using self-report questionnaires and the DASS-21 screening tool rather than structured clinical diagnostic interviews, increasing susceptibility to reporting bias and limiting the ability to establish definitive psychiatric diagnoses. Finally, important confounders were not fully captured (*e.g.*, detailed medication adherence, comorbid medical and psychiatric illnesses, and lifestyle factors), so the possibility of residual confounding remains.

## CONCLUSION

Research findings are still mixed on the best way to identify mental health symptoms and the factors that predispose young adults with hypertension. More evidence is needed to understand how strongly these problems affect quality of life and how best to address them in routine hypertension care. In this study, mental health problems were common among young hypertensive patients and were linked to a poorer quality of life. These results suggest that clinicians should not focus solely on blood pressure, but should also include mental health assessment as a regular part of care to support patients’ overall well-being.

## Figures and Tables

**Table 1:** Characteristics demographics.

**Demographic Variable**	**Category**	**Frequency (n)**	**Percentage**
Age (years)	Mean ± SD	33 ± 10.14	-
-	Range	18 – 45	-
Gender	Male	457	57.8
-	Female	334	42.2
City of Residence	Yogyakarta	365	46.1
-	Padang	201	25.4
-	Medan	200	25.3
-	Others	25	3.2
Ethnicity	Javanese	397	50.2
-	Minangkabau	210	26.5
-	Batak	106	13.4
-	Others	78	9.9
Marital Status	Married	550	69.5
-	Single	204	25.8
-	Divorced (living)	24	3.0
-	Divorced (deceased)	13	1.6
Education Level	Doctorate Degree	2	0.3
-	Master’s Degree	28	3.5
-	Bachelor’s Degree	220	27.8
-	Diploma’s Degrees	67	8.5
-	High School or Equivalent	394	49.8
-	Junior High School or Equivalent	59	7.5
-	Elementary School	18	2.3
-	No School	3	0.4
Occupation	Professionals	143	18.1
-	Self-Employed	117	14.8
-	Daily Labor	66	8.3
-	Civil Servant	62	7.8
-	Students	120	15.2
-	Unemployed	170	21.4
-	Others	113	14.2

**Table 2:** Correlation between WHOQOL-BREF domain with hypertension.

**WHOQOL-BREF Domain**	**Correlation (r)**	**95% CI**	***p*-value**
Physical Health	-0.35	-0.41 to -0.29	<0.001
Psychological Health	-0.40	-0.46 to -0.34	<0.001
Social Relationships	-0.25	-0.32 to -0.18	<0.001
Environment	-0.30	-0.36 to -0.24	<0.001
Overall Quality of Life	-0.45	-0.51 to -0.39	<0.001

**Table 3:** Correlation between DASS-21 domains and hypertension.

**DASS-21 domain**	**Correlation (r)**	**95% CI**	***p*-value**
Anxiety	-0.42	–0.48 to –0.36	<0.001
Depression	-0.38	–0.44 to –0.32	<0.001
Stress	-0.30	–0.36 to –0.24	<0.001

## Data Availability

The data and supportive information is available within the article.

## LIST OF ABBREVIATIONS

ACE: Adverse Childhood Experience
BMI: Body Mass Index
BP: Blood Pressure
CI: Confidence Interval
CIOMS: Council for International Organizations of Medical Sciences
CVD: Cardiovascular Disease
DASS-21: Depression Anxiety Stress Scale–21
r: correlation coefficient
SD: Standard Deviation
WHO: World Health Organization
WHOQOL-BREF: World Health Organization Quality of Life Instrument–BREF
